# Genetic Polymorphism Characteristics of *Brucella canis* Isolated in China

**DOI:** 10.1371/journal.pone.0084862

**Published:** 2014-01-23

**Authors:** Dongdong Di, Buyun Cui, Heng Wang, Hongyan Zhao, Dongri Piao, Lili Tian, Guozhong Tian, Jingli Kang, Xiang Mao, Xiaojun Zhang, Pengfei Du, Lin Zhu, Zhuo Zhao, Lingling Mao, Wenqing Yao, Pingyuan Guan, Weixing Fan, Hai Jiang

**Affiliations:** 1 Laboratory of Zoonoses, China Animal Health and Epidemiology Center, MOA, Qingdao, China; 2 State Key Laboratory for Infectious Disease Prevention and Control, Collaborative Innovation Center for Diagnosis and Treatment of Infectious Diseases, National Institute for Communicable Disease Control and Prevention, Beijing, China; 3 Laboratory of Endemic and Parasitic Diseases Control and Prevention, Hangzhou Center for Disease Control and Prevention, Hangzhou, China; 4 College of Veterinary Medicine, Nanjing Agricultural University, Nanjing, China; 5 Jining District Animal Disease Control Center, Jining District Agriculture and Animal Husbandry Bureau, Wulanchabu, China; 6 Liaoning Center for Disease Control and Prevention, Shenyang, China; 7 College of Veterinary Medicine, Inner Mongolia Agricultural University, Hohhot, China; East Carolina University School of Medicine, United States of America

## Abstract

In China, brucellosis is an endemic disease typically caused by *Brucella melitensis* infection (biovars 1 and 3). *Brucella canis* infection in dogs has not traditionally recognized as a major problem. In recent years however, brucellosis resulting from *Brucella canis* infection has also been reported, suggesting that infections from this species may be increasing. Data concerning the epidemiology of brucellosis resulting from *Brucella canis* infection is limited. Therefore, the purpose of this study was to assess the diversity among Chinese *Brucella canis* strains for epidemiological purposes. First, we employed a 16-marker VNTR assay (*Brucella* MLVA-16) to assess the diversity and epidemiological relationship of 29 *Brucella canis* isolates from diverse locations throughout China with 38 isolates from other countries. MLVA-16 analysis separated the 67 *Brucella canis* isolates into 57 genotypes that grouped into five clusters with genetic similarity coefficients ranging from 67.73 to 100%. Moreover, this analysis revealed a new genotype (2-3-9-11-3-1-5-1:118), which was present in two isolates recovered from Guangxi in 1986 and 1987. Second, multiplex PCR and sequencing analysis were used to determine whether the 29 Chinese *Brucella canis* isolates had the characteristic BMEI1435 gene deletion. Only two isolates had this deletion. Third, amplification of the *omp25* gene revealed that 26 isolates from China had a T545C mutation. Collectively, this study reveals that considerable diversity exists among *Brucella canis* isolates in China and provides resources for studying the genetic variation and microevolution of *Brucella*.

## Introduction

Brucellosis, caused by various species of the Gram-negative bacterium *Brucella*, continues to be one of the most serious zoonotic diseases for humans and animals throughout the world [1]. In recent years, the number of published reports describing brucellosis resulting from *B. canis* infection has increased [2]–[6]. Although, *B. canis* infection has not been the major cause of brucellosis in China [7]–[10], recent outbreaks in Beijing and other provinces suggest that this infection may be on the rise [8]. In order to effectively prevent this disease, it is important to identify what strain of *Brucella* has caused the infection. However, most molecular subtyping tools and “classical biotyping” methods lack sufficient discriminatory power for epidemiological investigations. Thus, effective molecular subtyping tools capable of reproducibly distinguishing differences between strains must be implemented.

The *Brucella* genus is genetically conserved [11]–[13], making rapid and accurate identification of species in the genus difficult. For instance, distinguishing between the species *B. suis* and *B. canis* has been particularly challenging [14]–[20]. This difficulty is due to the fact that few genetic polymorphisms discriminate between these two species at the molecular level as well as *B. canis* being a clonal lineage that arose from *B. suis* [21]. Several studies have optimized PCR based tests to address this issue [22]. For example, three multiplex PCR assays, a Bruce-ladder, a 19-primer multiplex PCR, and a new Bruce-ladder multiplex PCR assay called “v2.0 multiplex PCR” have been developed to characterize *Brucella* isolates at the species level [21], [23], [24]. More recently, multi-locus variable-number tandem-repeat analysis (MLVA) has been confirmed as a useful tool for identifying and genotyping *Brucella* isolates, and the data have been used for epidemiological investigations [25]–[28].

Bruce-ladder analysis generally performs well and has been recommended by the World Organization for Animal Health (OIE) as a rapid and simple one-step molecular test for identification and typing of *Brucella* species [24]. The 19-primer multiplex PCR assay was used to differentiate isolates of the genus *Brucella* at the species and biovar levels [21]. However, routine and widespread use of these two multiplex PCR typing tools has revealed that they misidentify a substantial proportion of *B. canis* isolates as *B. suis* [21], [29], [30]. The primary reason for this misclassification is the selection of the BMEI1435 gene as a marker for *B. canis*. This gene was originally chosen because it was thought to be naturally deleted in isolates of *B. canis* and would thus yield a smaller PCR product than other *Brucella* species containing this gene [31]; however, the BMEI1435 gene is not deleted in all *B. canis* isolates. Therefore, a substantial proportion of *B. canis* isolates cannot be correctly identified using this approach.

In this report, we present both *Brucella* MLVA-16 and PCR data from Chinese *B. canis* isolates recovered from brucellosis outbreaks from different geographical origins. The aims of this study were to twofold. Firstly, we sought to assess the performance of MLVA-16 molecular typing assay applied to Chinese *B. cani*s isolates and assess the diversity among *B. canis* strains for epidemiological purposes. Secondly, we wanted to evaluate the genetic relationship between Chinese *B. canis* strains and exogenous strains. We compared the results of Chinese *B. canis* isolates identified by deletion of the BMEI1435 gene, 19-primer multiplex PCR, Bruce-ladder PCR, and Bruce-ladder v2.0. In addition, because the *omp25* gene can be used to distinguish *B. canis* from other *Brucella* species [11], [32]–[34], we employed SNP-based typing using the *omp*25 gene as an alternative target.

## Materials and Methods

### Strains

A collection of *B. canis* isolates from infected dogs was obtained from various locations in China (including ten outbreak isolates from Beijing in 2011) and characterized using classical biotyping [35] (Table 1). Growth and harvesting of *Brucella* cells and bacterial DNA extraction were performed as previously described [23], [36]. All isolates were further examined using SNP typing, Bruce-ladder v2.0, 19-primer multiplex PCR (*B. canis* specific primers only) and Bruce-ladder. SNP-based typing to identify *Brucella* isolates at the species level was carried out as described by Gopaul *et al.* [32]. Bruce-ladder v2.0 was performed according to the method of Lopez-Goni *et al.* [24]. The 19-primer multiplex PCR was performed as described by Huber *et al.* [21]. Bruce-ladder was performed as described by Garcia-Yoldi *et al.* [23].

**Table 1:** *Brucella canis* isolates and characterization using different molecular typing methods.

Isolate^1^^,^ 2	Biotype	SNP typing	Bruce-ladder v2.0	19-primer multiplex PCR^a^	Bruce-ladder	Deletion^b^	Mutation^c^	Year	Place
*B. canis* RM6/66^1^	*B. canis*	*B. canis*	*B. canis*	*B. canis*	*B. canis*	Yes	T	1966	USA
*B. canis* 1124^1^	*B. canis*	*B. canis*	*B. canis*	*B. canis*	*B. canis*	Yes	T	2010	Inner Mongolia
*B. canis* 233^1^	*B. canis*	*B. canis*	*B. canis*	*B. canis*	*B. canis*	Yes	T	1986	Xinjiang
*B. canis* 231^1^	*B. canis*	*B. canis*	*B. canis*	Atypical *B. canis*	*B. suis*	No	T	1983	Shanghai
*B. canis* 232^1^	*B. canis*	*B. canis*	*B. canis*	Atypical *B. canis*	*B. suis*	No	C	1986	Jiangsu
*B. canis* 235^1^	*B. canis*	*B. canis*	*B. canis*	Atypical *B. canis*	*B. suis*	No	C	1986	Guangxi
*B. canis* 236^1^	*B. canis*	*B. canis*	*B. canis*	Atypical *B. canis*	*B. suis*	No	C	1986	Zhejiang
*B. canis* 237^1^	*B. canis*	*B. canis*	*B. canis*	Atypical *B. canis*	*B. suis*	No	C	1986	Guangxi
*B. canis* 239^1^	*B. canis*	*B. canis*	*B. canis*	Atypical *B. canis*	*B. suis*	No	C	1987	Henan
*B. canis* 240^1^	*B. canis*	*B. canis*	*B. canis*	Atypical *B. canis*	*B. suis*	No	C	1987	Jiangxi
*B. canis* 241^1^	*B. canis*	*B. canis*	*B. canis*	Atypical *B. canis*	*B. suis*	No	C	1987	Shandong
*B. canis* 243^1^	*B. canis*	*B. canis*	*B. canis*	Atypical *B. canis*	*B. suis*	No	C	1987	Anhui
*B. canis* 244^1^	*B. canis*	*B. canis*	*B. canis*	Atypical *B. canis*	*B. suis*	No	C	1987	Guangxi
*B. canis* 245^1^	*B. canis*	*B. canis*	*B. canis*	Atypical *B. canis*	*B. suis*	No	C	1988	Hubei
*B. canis* 247^1^	*B. canis*	*B. canis*	*B. canis*	Atypical *B. canis*	*B. suis*	No	C	1988	Jiangxi
*B. canis* 249^1^	*B. canis*	*B. canis*	*B. canis*	Atypical *B. canis*	*B. suis*	No	C	1988	Liaoning
*B. canis* 251^1^	*B. canis*	*B. canis*	*B. canis*	Atypical *B. canis*	*B. suis*	No	C	1989	Hebei
*B. canis* RU^1^	*B. canis*	*B. canis*	*B. canis*	Atypical *B. canis*	*B. suis*	No	C	2007	Jiangsu
*B. canis* XUE1^1^	*B. canis*	*B. canis*	*B. canis*	Atypical *B. canis*	*B. suis*	No	C	2010	Inner Mongolia
*B. canis* LI^2^	*B. canis*	*B. canis*	*B. canis*	Atypical *B. canis*	*B. suis*	No	C	2010	Liaoning
*B. canis* BJ-03^1^	*B. canis*	*B. canis*	*B. canis*	Atypical *B. canis*	*B. suis*	No	C	2011	Beijing
*B. canis* BJ-10^1^	*B. canis*	*B. canis*	*B. canis*	Atypical *B. canis*	*B. suis*	No	C	2011	Beijing
*B. canis* BJ-13^1^	*B. canis*	*B. canis*	*B. canis*	Atypical *B. canis*	*B. suis*	No	C	2011	Beijing
*B. canis* BJ-15^1^	*B. canis*	*B. canis*	*B. canis*	Atypical *B. canis*	*B. suis*	No	C	2011	Beijing
*B. canis* BJ-18^1^	*B. canis*	*B. canis*	*B. canis*	Atypical *B. canis*	*B. suis*	No	C	2011	Beijing
*B. canis* BJ-19^1^	*B. canis*	*B. canis*	*B. canis*	Atypical *B. canis*	*B. suis*	No	C	2011	Beijing
*B. canis* BJ-38^1^	*B. canis*	*B. canis*	*B. canis*	Atypical *B. canis*	*B. suis*	No	C	2011	Beijing
*B. canis* BJ-72^1^	*B. canis*	*B. canis*	*B. canis*	Atypical *B. canis*	*B. suis*	No	C	2011	Beijing
*B. canis* BJ-73^1^	*B. canis*	*B. canis*	*B. canis*	Atypical *B. canis*	*B. suis*	No	C	2011	Beijing
*B. canis* BJ-89^1^	*B. canis*	*B. canis*	*B. canis*	Atypical *B. canis*	*B. suis*	No	C	2011	Beijing

a : *B.canis* specific primers only;

b : Characterization of *B. canis* BMEI1435 gene deletion (related to the genome of *B. melitensis* 16M, AE008917).

c : The mutation of *omp*25 gene at position 545 (SNP position related to the genome of *B. abortus* 9-941, GenBank ID: AE017223).

1 : isolated from dog.

2 : isolated from human.

### Characterization of *B. canis* BMEI1435 gene deletion

In order to characterize deletion of the BMEI1435 gene, primers BMEI 1434F (5′-GCCAGCCACAGGATCAGGTGAT-3′) and BMEI 1436R (5′- GGATCCGTTCGTTTCGCTCG-3′) [31] were used to amplify the BMEI1435 gene. The length of the product was 1674 bp (containing the BMEI1435 gene) or 607 bp (the BMEI1435 gene deletion). Products were then separated by agarose gel electrophoresis and sequenced.

### Characterization of *B. canis* in *omp*25 gene

Primers were designed based on the sequence of the *omp*25 gene from *B. melitensis* 16M (*omp*25-F: CATGGGCGGTTTACTC; *omp*25-R: CGGCCAGATCATAGTTC). The *omp*25 gene of 29 Chinese *B. canis* isolates was amplified using primers *omp*25-F and *omp*25-R. The length of the product was 652 bp, and the products were amplified and sequenced by the Sanger method. These *omp*25 gene sequences were aligned with *B. canis* RM6/66 and 13 other *B. canis omp*25 gene sequences obtained from GenBank.

### Characterization of *B. canis* by MLVA genotyping

MLVA was performed as described previously with the following modifications [37], [38]. The 16 primer pairs were divided into three groups: MLVA-8 (eight loci including bruce06, bruce08, bruce11, bruce12, bruce42, bruce43, bruce45 and bruce55), panel 2A (three loci including bruce18, bruce19 and bruce21), and panel 2B (five loci including bruce04, bruce07, bruce09, bruce16 and bruce30). Forward primers of the panel 2A and 2B loci were labeled with one of four 5′-fluorescent labels (6-FAM, ROX, HEX, or TAMRA). These primers were obtained from Shenggong Biosciences, Inc., (Shanghai, China). After an initial denaturation at 94°C for 3 min, the PCR conditions were as follows: 30 cycles of denaturation at 94°C for 30 seconds, annealing at 60°C for 30 seconds, and extension at 72°C for 50 seconds. Five microliters of the panel one loci amplification products were loaded in to 2% agarose gels containing ethidium bromide (0.5 µg/ml), visualized under UV light, and photographed. To determine the number of repeats from the sample products, PCR products were purified and directly sequenced using an ABI Prism Big Dye Terminator (v3.1) cycle sequencing ready reaction kit (v5.0; Applied Biosystems, Foster City, CA, USA). The PCR products of these samples were then sequenced and compared to the sequence of *B. melitensis* 16M. PCR products of panel 2A and 2B loci were denatured and resolved by capillary electrophoresis on an ABI Prism 3130 automated fluorescent capillary DNA sequencer (Applied Biosystems). Fragments were sized by comparison to a ROX (carboxy-X-rhodamine)-labeled molecular ladder (MapMaker 1000; BioVentures Inc., Murfreesboro, TN, USA) with GeneMapper software version 4.0 (Applied Biosystems). Appropriate VNTR designations of the fragments were assigned based on size calling through internal software binning capabilities and the corresponding repeat copy numbers of *B. melitensis* 16M.

### Analysis of MLVA data

All data were analyzed using BioNumerics software version 5.1 (Applied Maths, Sint-Martins-Latem, Belgium). Cluster analysis was based on the categorical coefficient and the unweighted pair group method using arithmetic averages (UPGMA). The genotypes were then compared using the web-based *Brucella* 2012 MLVA database (http://mlva.u-psud.fr/). The genotyping data can be found in the supplementary data (Table S1).

## Results

### Molecular typing

Given that routine multiplex PCR typing tools often misidentify a substantial proportion of *B. canis* isolates as *B. suis*, 29 Chinese *B. canis* isolates were identified using classical biotyping method, and then further examined them by SNP typing, Bruce-ladder v2.0, 19-primer multiplex PCR (*B. canis* specific primers only) and Bruce-ladder. Consistent with phenotypic typing, SNP genotyping and Bruce-ladder v2.0 identified all 29 isolates as *B. canis*. However, only two of the 29 *B. canis* isolates (1124 and 233) were identified as *B. canis* by Bruce-ladder and 19-primer multiplex PCR. The other 27 isolates were identified as *B. suis* (Table 1). In addition, using the *B. canis* specific primers from the 19-primer multiplex PCR assay reported by Huber *et al.* (2009) [21], we amplified products of 836 and 1903 base pairs. These two products represented *B. canis* isolates lacking and containing the BMEI1435 gene, respectively. To assess the correlation between *B. canis* and BMEI1435 gene deletion, these 29 isolates were then analyzed for the presence of the BMEI1435 gene by PCR amplification. The BMEI1435 gene was only deleted in isolates 1124 and 233, the same two isolates identified as *B. canis* by Bruce ladder and 19-primer multiplex (Table 1). These data suggest that SNP typing and Bruce-ladder v2.0 assay are more effective methods for identifying *B. canis* and that not all *B. canis* strains have a deletion of the BMEI1435 gene.

### Characterization of the *omp*25 gene in *B. canis*

In addition to the BMEI1435 marker, the *omp*25 gene has also been used to distinguish between species of *Brucella*. Therefore, the *omp*25 gene of the 29 Chinese *B. canis* isolates was amplified a 625 bp product. Twenty-six of 29 *B. canis* isolates had an *omp*25 T545C mutation, while the other three had no mutations (Table 1).

### MLVA analysis of 29 Chinese *B. canis* isolates

We next sought to identify a more effective molecular tool for detecting the genetic variation of *Brucella* isolates. Thus, we employed a *Brucella* MLVA-16 assay to assess the diversity and epidemiological relationship between 29 *B. canis* isolates from diverse locations throughout China. This method provided a high discriminatory power (HGDI of 0.956) with a genetic similarity coefficient ranging from 69.46 to 100% (Table 2). The HGDI values were determined at seven loci: Bruce 04, 07, 09, 11, 16, 18 and 55 (Table 2). Bruce09 was identified as having the highest diversity overall. In addition, MLVA-16 analysis distributed the 29 Chinese *B. canis* isolates into three MLVA-8 genotypes (2-3-9-11-3-1-5-2, 2-3-8-11-3-1-5-2 and 2-3-9-11-3-1-5-1) and 21 MLVA-16 genotypes (Table 2). A vast majority of Chinese *B. canis* isolates belonged to MLVA-8 genotype 3 (2-3-9-11-3-1-5-2); however, a new genotype 118 (2-3-9-11-3-1-5-1) was detected in two isolates from Guangxi recovered in 1986 and 1987.

**Table 2:** Hunter-Gaston Diversity Index (HGDI) for the 67 *Brucella canis* isolates.

	29 Chinese *B. canis* isolates			67 *B. canis* strains		
Locus	No. of alleles	HGDI^a^	CI 95%^b^	No. of alleles	HGDI^a^	CI 95%^b^
MLVA-16	21	0.956	0.906–1.000	57	0.991	0.981–1.000
MLVA-8	3	0.310	0.107–0.513	4	0.197	0.071–0.323
Bruce06	1	0.000	0.000–0.210	1	0.000	0.000–0.101
Bruce08	1	0.000	0.000–0.210	1	0.000	0.000–0.101
Bruce11	2	0.192	0.016–0.368	3	0.143	0.031–0.255
Bruce12	1	0.000	0.000–0.210	1	0.000	0.000–0.101
Bruce42	1	0.000	0.000–0.210	1	0.000	0.000–0.101
Bruce43	1	0.000	0.000–0.210	1	0.000	0.000–0.101
Bruce45	1	0.000	0.000–0.210	1	0.000	0.000–0.101
Bruce55	2	0.133	0.000–0.292	2	0.059	0.000–0.135
Panel 2A	3	0.616	0.519–0.712	4	0.532	0.423–0.641
Bruce18	3	0.616	0.519–0.712	3	0.454	0.343–0.565
Bruce19	1	0.000	0.000–0.210	2	0.114	0.014–0.214
Bruce21	1	0.000	0.000–0.210	1	0.000	0.000–0.101
Panel 2B	21	0.956	0.906–1.000	56	0.991	0.980–1.000
Bruce04	5	0.547	0.362–0.732	6	0.697	0.626–0.768
Bruce07	7	0.714	0.587–0.841	8	0.624	0.528–0.720
Bruce09	10	0.860	0.790–0.929	12	0.887	0.861–0.913
Bruce16	7	0.825	0.747–0.903	7	0.842	0.818–0.866
Bruce30	1	0.000	0.000–0.210	1	0.000	0.000–0.101

a : Hunter and Gaston index.

b : Precision of the diversity index, expressed as 95% upper and lower boundaries.

**Figure 1:**
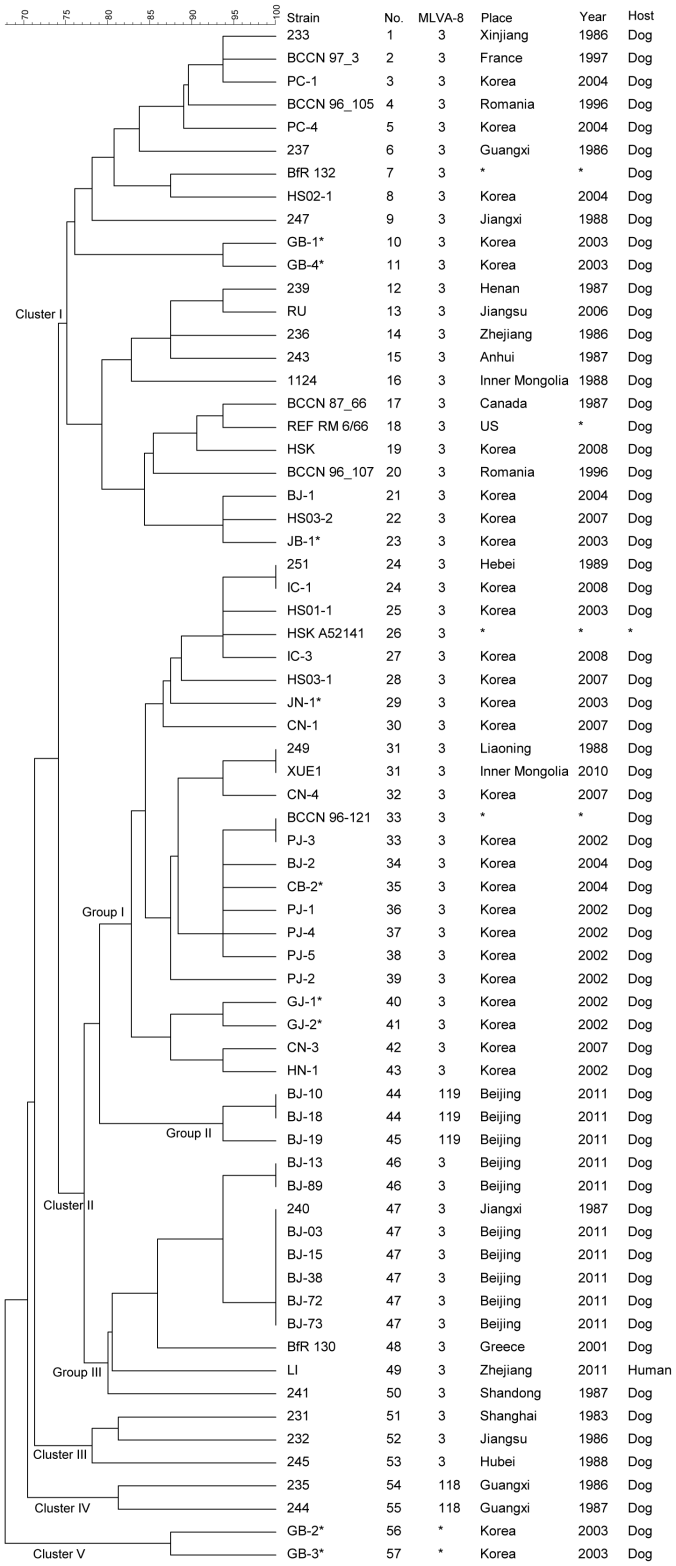
Dendrogram derived from the MLVA-16 genotyping assay. This dendrogram illustrates the various relationships among the 29 Chinese *B. canis* isolates and the 38 non-Chinese *B. canis* strains. In the columns, the following data for isolates are indicated: Strain: laboratory identifier of isolate in which the DNA extraction was performed, No. genotype numbering, Place/Year: country and year of isolation (when known), MLVA-8: genotype numbers associated with the genotypes corresponding to each isolates in the database.

We next sought to extend this analysis to isolates from regions outside of China. MLVA-16 analysis of 67 *B. canis* strains (29 from China, and 38 from the *Brucella*2012MLVA database and a report by Kang, SI *et al.* in 2011) provided a high discriminatory power (HGDI of 0.991) with a genetic similarity coefficient ranging from 67.73 to 100% (Figure 1). These 67 *B. canis* strains were distributed into four MLVA-8 genotypes and 57 MLVA-16 genotypes (Table 2), and separated into five major clusters. Cluster I covered 23 MLVA-16 genotypes comprising eight isolates recovered from China (Xinjiang, Guangxi, Jiangxi, Henan, Jiangsu, Zhejiang, Anhui and Inner Mongolia), 15 from other countries (US, Canada, Romania, France, Greece, Korea and one unknown source) and the RM6/66 reference strain. All Cluster I isolates shared the MLVA-8 genotype 3.

Cluster II contained 37 *B. canis* isolates (16 from China, 18 from Korea, one from Greece and two from unknown sources) grouped in 27 MLVA-16 genotypes. All Cluster II isolates shared the MLVA-8 genotype 3, with only three exceptions, corresponding to the MLVA-8 genotypes 119. In addition, the 37 isolates of Cluster II were separated into three groups. Group I contained 23 isolates, with only six of the Chinese *B. canis* isolates included. Interestingly, three pairs of isolates with the same MLVA-16 pattern (Hebei [1989] and Korea [2008]; Inner Mongolia [2010] and Liaoning [1988]; and Korea [2002] and one unknown source) were clustered into three MLVA-16 genotypes (24, 31 and 33) in Cluster II Group I. Group II consisted of only three outbreak isolates recovered from Beijing, and separated into two MLVA-16 genotypes (44 and 45). The other five Beijing outbreak isolates (2011) and one of the Jiangxi isolates (1987) clustered to one MLVA-16 genotype (47) in Group III. In addition, two other isolates from a Beijing outbreak (2011) grouped in one MLVA-16 genotype (46) in Group III.

Cluster III contained three isolates from Shanghai, Jiangsu and Hebei. Cluster IV contained two Guangxi isolates (MLVA-8: 118). Cluster V was comprised of two Korea isolates.

## Discussion

*B. canis* was first isolated in China from domestic and imported beagles in 1984 [7]; however, the epidemiological characteristics of *B. canis* infection are limited. Although *B. canis* infection has not been a major national concern, recent reports of *B. canis* infections in Beijing and other provinces highlight the significance of defining the epidemiological characteristics of this organism [8]. Currently, the MLVA-16 assay is being used to analyze epidemiological correlations between various strains. This method can also be used to track the geographic origin by comparing genetic patterns of endogenous strains with foreign isolates [2], [27], [28]. In this study, 29 Chinese *B. canis* isolates from different locations were analyzed by MLVA-16 assay, and compared with 38 *B. canis* isolates from other countries.

This study revealed that MLVA-16 genotype 31 (249 from Liaoning in 1988 and XUE1 from Inner Mongolia in 2010) was detected in different provinces with over twenty years between outbreaks. In addition, five of ten Beijing *B. canis* outbreak isolates (in 2011) and the Jiangxi isolate (in 1987) belonged to MLVA-16 genotype 47 [8]. These data suggest that *B. canis* strains might spread throughout China. Similarly, when compared with foreign strains, the MLVA-16 pattern of the domestic *B. canis* isolate 251 was identical with a Korean strain (IC-1). This suggests that poor importation quarantine may account for a subset of *B. canis* infections. Together, these data suggest that the MLVA-16 assay can be applied to long-term surveillance, and investigation of *B. canis* origins and epidemiological relatedness. This information will be valuable to establish strategies for a nationwide survey of *B. canis* infections and potential human exposure risks.

Another layer of *B. canis* diversity is the presence or absence of the BMEI1435 gene. Garcia-Yoldi *et al.* reported that 11 *B. canis* isolates (from USA, Mexico, Argentina, Germany, South Africa and Japan) contained the BMEI1435 gene; however this gene was not present in 13 different *B. canis* isolates (from USA, Germany, Peru and United Kingdom) [29]. Similarly, Huber *et al.* reported that nine *B. canis* isolates contained the BMEI1435 gene, but this gene was absent in six *B. canis* isolates (isolated from dogs and one unknown source) [21]. In the present study, sequence analysis of the BMEI1435 gene region was performed after amplification using the primers BMEI1434F and BMEI1436R. Compared to the reference train RM6/66, the BMEI1435 gene was absent in only two of the 29 *B. canis* isolates (1124 and 233) analyzed. The remaining 27 isolates contained the BMEI1435 gene, which was identical to *B. canis* HSK A52141 (CP003174). These data reveal that *B. canis* isolates with and without the BMEI1435 gene exist in China.

Huber *et al.* also reported that a PCR product of 887 bp (*B. canis* isolates lacking the BMEI1435 gene, including *B. canis* reference strain RM6/66) or a faint amplicon of 1863 bp (*B. canis* isolates containing the BMEI1435 gene) were amplified using *B. canis* specific primers [21]. In this study, using the *B. canis* specific primers reported by Huber *et al.* [21], PCR products of 836 bp and 1903 bp were obtained. To account for this discrepancy, we performed sequence analysis. Alignment of the 1903 bp product with *B. canis* HSK A52141 (CP003174) revealed 100% sequence identity, and alignment of the 1903 bp product with the sequence of the *B. canis* reference strain RM6/66 revealed that strain RM6/66 had the expected deletion (1067 bp). A representative sequence was submitted to GenBank (Accession number KC572141). A similar analysis of the 836 bp product revealed 100% identity with the *B. canis* reference sequence from strain RM6/66. Sequences of this product have also been submitted to GenBank (Accession number KC572142).

We also sought to identify various *B. canis* strains by sequencing the *omp25* gene. Gene sequences from the 29 Chinese *B. canis* isolates were aligned with *omp*25 gene sequences from *B. canis* RM6/66 and other strains obtained from GenBank. This analysis revealed that the *omp*25 gene sequences of 26 Chinese *B. canis* isolates have the *omp25* T545C mutation, and this mutation is consistent with three *B. canis* isolates isolated from Germany and South Africa (AM695188, AM695179 and AM695170). Like *B. canis* RM6/66, the *omp25* gene position T545 of the remaining three isolates (1124, 233 and 231) was not mutated. The *omp*25 gene sequence also correlated to the presence of the BMEI1435 gene. Two of the *B. canis* isolates without the *omp*25 T545C mutation (1124 and 233) lacked the BMEI1435 gene like *B. canis* RM6/66. Twenty-six of the 27 remaining isolates were strains with the *omp*25 T545C mutation also had the BMEI1435 gene. These data revealed that the base at position 545 of *the omp*25 gene correlated well with the presence of the BMEI1435 gene. One exception was the isolate 231. This isolate did not have the typical *B. canis* deletion of BMEI1435 gene, but had the *omp*25 T545C mutation. Further experimentation is required to fully elucidate this relationship.

## Conclusions

SNP genotyping and Bruce-ladder 2.0 assay were able to sufficiently resolve *B. canis* and *B. suis* species. *B. canis* isolates with and without the BMEI1435 gene were present in China. In addition, a point mutation in the *omp*25 gene position 545 correlated with the presence of the BMEI1435 gene. This study reveals that the MLVA-16 assay is an effective molecular tool for detecting genotype distribution of *Brucella* isolates from endemic and non-endemic areas, and that considerable diversity among *B. canis* isolates in China.

## Supporting Information

Table S1 **MLVA-16 genotypes for 67** ***B. canis*** **strains.**
(XLS)
